# Supplementary material for: Patterns of Intron Gain and Loss in Fungi
Source: PLoS Biol. 2004 Nov 30;2(12):e422. doi: 10.1371/journal.pbio.0020422 (PMC532390; doi:10.1371/journal.pbio.0020422)
Supplement: Table S1 — Also available at http://genes.mit.edu/NielsenEtAl/. (4.3 MB ZIP). [file pbio.0020422.st001.zip › NielsenEtAl/html/713.html]

AN5482.1.NCU09269.1.MG09952.1.FG01099.1


```
 CLUSTAL W (1.82) Multiple Sequence Alignments - Introns Inserted


Sequence 1: NCU09269.1	215 aa
Sequence 2: FG01099.1	216 aa
Sequence 3: AN5482.1	222 aa
Sequence 4: MG09952.1	208 aa
Alignment Length: 224 aa
Number Identitical Residues: 166 aa
Alignment Score (without introns) 6855


MG09952.1 	MAEAQQVPTFKLVL0T---------~-----~-TFVKRHLTGEFEKKYMATLGVEVHPLG
NCU09269.1	--MAAATPTFKLVL~VGDGGTG---~----K0TTFVKRHLTGEFEKKYMATLGVEVHPLG
FG01099.1 	-MAEQQTPTFKLVL~VGDGGTG---~----K0TTFVKRHLTGEFEKKYMATLGVEVHPLG
AN5482.1  	-MAEQQVPTFKLVL~VGDGGTGKAN1FLPSQ~TTFVKRHLTGEFEKKYIATLGVEVHPIK
          	      .******* .....:..:.   .:. :***************:*********: 

MG09952.1 	FET0NFGKIQFDVWDTAGQEKFGGLRDGYYINGQCGIIMFDVTSRITYKNVPNWHR1DLV
NCU09269.1	FST0NFGQIQFDVWDTAGQEKFGGLRDGYYINGQCGIIMFDVTSRITYKNVPNWHR1DLT
FG01099.1 	FTT0NFGQIQFDVWDTAGQEKFGGLRDGYYINGQCGIIMFDVTSRITYKNVPNWHR1DLV
AN5482.1  	FTT0NLGTIQFDVWDTAGQEKFGGLRDGYYINGQCGIIMFDVTSRITYKNVPNWHR1DLV
          	* * *:* ************************************************ **.

MG09952.1 	RVCENIPIVLCGNKVDVKERKVKAKTITFHRKKNLQYYDISAKSNYNFEKPFLWLGRKLV
NCU09269.1	RVCENIPIVLCGNKVDVKERKVKAKTITFHRKKNLQYYDISAKSNYNFEKPFLWLARKLV
FG01099.1 	RVCENIPIVLCGNKVDVKERKVKAKTITFHRKKNLQYYDISAKSNYNFEKPFLWLARKLV
AN5482.1  	RVCENIPIVLCGNKVDVKERKVKAKTITFHRKKNLQYYDISAKSNYNFEKPFLWLARKLV
          	*******************************************************.****

MG09952.1 	GNPGL0EFVAAPALAPPTAEVTAEQAAAYEKDLADATRAPLPDD-DDEDF
NCU09269.1	GNNAL0EFVAAPALAPPTAVVDQELMEKYRAEMDEAAQMPLPNEEDDDDL
FG01099.1 	GNPQL0EFVAAPALAPPTAQVDEKLLEEYRKEMDEAAAMPLPGELSDDDL
AN5482.1  	GNASL0EFVAAPALAPPEVQVDATLMQQYSDEMAAAANQPLPDE-DDADL
          	**  * *********** . *       *  ::  *:  ***.: .* *:
```
